# Hospital Treatment as an Additional Benefit

**Published:** 1921-09-10

**Authors:** 


### The Hospital

The Workers' Newspaper of Administrative Medicine and Institutional Life, Administration, National Insurance and Health.

No. 1840, Vol. LXX. SATURDAY, SEPTEMBER 10, 1921. PRICE TWOPENCE

### HOSPITAL TREATMENT AS AN ADDITIONAL BENEFIT.

The British Hospitals Association has issued an important circular to its members and also to its Regional Committees in regard to hospital treatment as an approved society additional benefit. The object of the circular is to explain exactly what the approved societies expect in return for payments made by them. In the meantime, it is desirable that all hospitals should keep a careful record of patients who are members of approved societies, and in fact such a record is presumed already in the almoner's or inquiry department of many hospitals. The British Hospitals Association has, therefore, drafted a scheme to enable an approved society to make a fair distribution to hospitals from a fund formed by a society to assist its members to obtain hospital treatment. This scheme is now offered for consideration, and it is important that the honorary secretaries of the Regional Committees should call meetings to consider the scheme as soon as possible. The main points of the scheme are as follows: Since subscriptions from the approved societies to particular hospitals will not satisfy the requirements of the Ministry of Health, and each member of the societies must have a reasonable chance to participate, payment in respect of each member seems to be the only satisfactory method, if a simple way can be found to determine the amount of the subscription for each person treated without involving expensive correspondence and delay. Proceeding on the basis that approved societies, with the necessary funds, can by way of additional benefit subscribe toward the cost of their members' maintenance in hospital, the scheme proposes that each society will set aside a certain sum for this purpose. That sum would be the amount to be distributed, and the object of the scheme is to provide a convenient method of allotting it to hospitals in a fair proportion according to the extent of the treatment which they provide for the societies' members.

It is, therefore, proposed that each society should apportion the amount to be distributed every quarter or half-year, or should invite the hospitals to send a list of the society's members who have received in-patient treatment with particulars of the membership and the duration, in weeks, of the treatment. Upon an agreed date, allowing the hospitals enough time to compile these statements, the society would ascertain the amount of the contributions to be paid to the different hospitals. "This sum would be arrived at by dividing the total sum to be distributed in the quarter or half-year by the total number of weeks' treatment claimed by the hospitals and multiplying the quotient by the number of weeks claimed by each separate hospital." While each hospital would, therefore, receive something in return for its treatment of every member of an approved society offering the additional benefit, each such society would "pay no more and no less than the sum which had been determined as the amount to be distributed."

This amount will be, of course, at the discretion of the societies, and the scheme also assumes that hospitals will require their in-patients to contribute according to their means. There can be little doubt that from both points of view the scheme is an attempt in the right direction, and the correspondence which has passed between the British Hospitals Association and the approved societies shows the difficulties which have had to be overcome. From the point of view of the societies, the contributions suggested represent an additional benefit, and must therefore bring some advantage not enjoyed by members of a society which do not contribute. From the point of view of the hospitals, urgency is the claim to priority of admission, and their problem is to reconcile this with the bestowal of some advantage upon patients who are members of societies providing "hospital benefit." The principle adopted under the scheme is for the hospi[illegible] first, as hitherto, to consider applications for treatment according to urgency, and then to consider, when assessing an in-patient's ability to pay toward the cost of his maintenance, whether the society to which he belongs already contributes.

Since the scheme, if generally adopted, is yet to begin as an experiment for one year, it would do little service to criticise it. An accommodation with the approved societies is much to be desired, even though in equity there are arrears to be made up by them. Experience will show how far the scheme is workable. The approved societies have to discover whether their members will allege that the scheme does not in fact confer an additional benefit. The hospitals, among other matters, have to learn how such benefit as they can confer on members of the contributing societies who become in-patients affects the contributions which many of these patients at present make.

The matter must be subjected to the test of experience. Meantime it should be remembered that one class of hospitals is likely to remain outside the scheme. Contributions to lying-in hospitals are not contemplated by the societies, presumably because a maternity benefit already exists, and, generally speaking, the lying-in hospitals have not made strong claims upon the approved societies. In some parts of the country, particularly in Scotland, there has been a lack of maternity cases, and hospitals with medical schools have been wishing for institutional treatment to be recommended more often. It seems clear at any rate that these societies prepared to adopt the new scheme experimentally will not contribute to lying-in hospitals. So far as we are aware at the time of writing, this limitation is not likely to jeopardise the scheme. The next meeting of the Council of the British Hospitals Association will be held towards the end of September, and by that time the general attitude of the hospitals to the new scheme will probably be known.

